# Towards clinical implementation of T2-weighted cine imaging for intrafraction drift correction workflows on the 1.5 Tesla magnetic resonance-linear accelerator

**DOI:** 10.1016/j.phro.2025.100859

**Published:** 2025-11-01

**Authors:** Lieke T.C. Meijers, Johannes C.J. de Boer, Jochem R.M. van de Voort van Zyp, Nicole G.P.M. Vissers, Reijer H.A. Rutgers, Eveline Alberts, Jasmijn M. Westerhoff, Alice M. Couwenberg, Marieke I. Snijder-van As, Stefano Mandija, Edwin Versteeg, Martijn P.W. Intven, Bas W. Raaymakers, Astrid L.H.M.W. van Lier

**Affiliations:** a Department of Radiation Oncology, University Medical Center Utrecht, the Netherlands; b Philips Healthcare, Best, the Netherlands

**Keywords:** MR-Linac, Intrafraction drift correction, Comprehensive motion management, Pelvic targets, T2-weighted cine

## Abstract

**Background and purpose:**

In magnetic resonance–linear accelerator (MR-Linac) treatments, balanced Turbo Field Echo (bTFE) cine imaging is used to monitor and correct intrafraction motion but provides limited contrast and is prone to artifacts. This study evaluates a T2-weighted Turbo Spin Echo (T2-TSE) cine sequence for intrafraction drift correction in pelvic targets and compares its performance with the bTFE-cine.

**Materials and methods:**

During 86 fractions in 52 patients with pelvic tumors, the standard bTFE-cine and the T2-TSE-cine images were acquired interleaved coronal and sagittal on the MR-Linac. Pre- and post-treatment T2-weighted 3D images were registered. Intrafraction motion, deviations between 3D-3D and 3D-2D registration, and jitter were compared. Visual grading scores and acoustic noise levels were assessed in both sequences.

**Results:**

The bTFE-cine shows in 26 cases > 1 mm between the 3D-3D and 3D-2D registration compared to 6 cases for the T2-TSE-cine in all directions, however these differences were not statistically significant. The jitter was higher for the bTFE-cine in prostate cases compared to T2-TSE-cine, but comparable or less for rectum and pelvic lymph node metastatic cases. The visual assessment indicates a high preference for the T2-TSE-cine regarding target, organs at risk (OAR) visualization and in terms of artifacts. The equivalent sound levels for the bTFE-cine and T2-TSE-cine were 110.7 and 100.1 dB(A), respectively.

**Conclusion:**

This study shows comparable tracking results, higher visual grading scores and lower acoustic noise levels of the T2-TSE-cine sequence over the bTFE-cine used for intrafraction drift correction workflows of pelvic targets on the MR-Linac.

## Highlights

• Integration of high-quality T2-cine images in magnetic resonance guided radiotherapy.
• Balanced- and T2-weighted-cine demonstrated comparable target tracking performance.
• T2 turbo spin echo cine imaging was consistently preferred by all observers.
• Acoustic noise reduced by 9.6 dB for T2-cine, enhancing patient comfort.

## Graphical abstract

**Figure:**
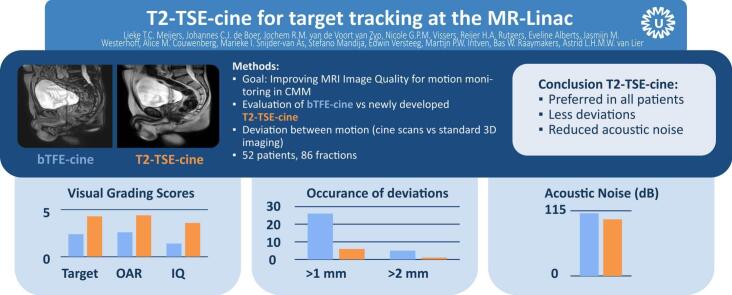

## 1 Introduction

The clinical introduction of magnetic resonance–linear accelerator (MR-Linac) systems has allowed treatment with improved visualization of pelvic target volumes (i.e. prostate, rectum and cervical cancers and pelvic lymph node oligometastases) and surrounding healthy tissues due to high soft tissue contrast [1,2]. Prostate and rectal cancers and pelvic lymph node oligometastases, further referred to as Lymph Node Metastasis (LNM), are the most commonly treated sites on the MR-Linac [3]. These target volumes are moving due to changes in bladder and rectum filling, overall relaxation, muscle tension and posture changes. This intrafraction motion is one of the biggest uncertainties in the treatment chain of radiotherapy [[4], [5], [6], [7], [8], [9]]. Prostate intrafraction motion on the MR-Linac has been reported by De Muinck Keizer et al. based on 3D imaging and 95 percent confidence intervals ranged between [-2.0 – 1.8] mm, [-3.8 – 4.1] mm and [-4.1 – 3.6] mm in Left-Right (LR), Anterior-Posterior (AP) and Superior-Inferior (SI) directions respectively for 590 fractions during 27 min [10]. Xiong et al. have reported prostate intrafraction motion for patients treated on the MR-Linac based on 2D imaging over 13 min in AP and SI direction of the clinical target volume (CTV) prostate of [-3.5 – 2.7] mm and [-2.9 – 4.2] mm respectively [11]. Therefore intrafraction motion corrections are required to further reduce planning target volume (PTV) margins for magnetic resonance imaging (MRI) guided prostate cancer radiotherapy.

Currently, MRI-guided treatments are moving towards hypo-fractionated treatments of 1–3 fractions with longer treatment times and therefore intrafraction motion corrections become even more relevant [[12], [13], [14]]. In our department a sub-fractionation scheme was implemented for primary prostate patients; halfway during beam-on time a new 3D image is acquired and the treatment plan is shifted according to the displacement of the CTV at that point in time, irrespective of whether intrafraction motion has occurred [15]. This allowed for a reduction of CTV to planning target volume (PTV) margins of 2–3 mm. However, the treatment plan should ideally be adjusted only when a clinically relevant shift persists for an extended period. The 0.35T MR-Linac system already allows treatment adaptation by applying a couch shift when the target moves outside predefined limits [11].

The latest 1.5T MR-Linac software allows intrafraction motion correction by shifting the treatment plan [16]. To measure, verify, and correct for the amount of intrafraction motion, good image quality is required. Currently, a 2D interleaved coronal and sagittal balanced Turbo Field Echo (bTFE) cine sequence is used for all treatments, including the pelvis. While bTFE-cine provides high signal-to-noise ratios, it differs in contrast from the T2-weighted 3D delineation images and shows image artifacts near air pockets [17,18]. Its high 5 Hz temporal resolution captures respiratory motion effectively but is unnecessary in pelvic targets where motion occurs on slower timescales [11,19]. Additionally, the sequence generates high acoustic noise due to gradient switching, which may cause considerable discomfort for patients during repeated treatments.

T2-weighted contrast is commonly used to define targets and organs at risk (OAR) in pelvic radiotherapy and could serve as a basis for a new cine sequence [[20], [21], [22], [23]]. This study evaluated a T2-weighted cine MRI sequence for intrafraction motion of pelvic targets and compared it to bTFE-cine at the MR-Linac in terms of tracking accuracy, visual grading, and acoustic noise. We hypothesized that the new sequence would provide superior image quality and increase user confidence compared to the existing bTFE-cine.

## 2 Materials and methods

### 2.1 Motion management

The newest implementation of the Unity MR-Linac (Elekta AB, Stockholm, Sweden) workflow is the use of Comprehensive Motion Management (CMM) which gives the possibility to correct for intrafraction motion during treatment using interleaved 2D bTFE-cine images. It has integrated two different modules: 1) it can deliver gated treatments for tumors that move due to respiratory motion and 2) it can compensate for drift motion using Baseline Shift (BLS) plan adaptation in which case the multi-leaf collimator (MLC) position is re-optimized.

### 2.2 Protocol development

**Fig. 1:**
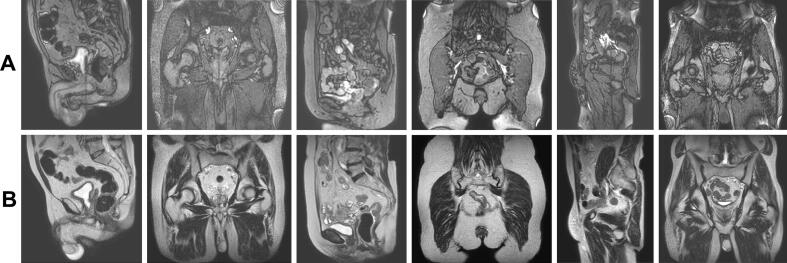
Representative image quality of bTFE-cine (A) and T2-TSE-cine (B) acquisitions in patients treated at the MR-Linac for prostate cancer, rectal cancer and oligolymphnode metastases. The upper image in panel A corresponds to the same patient as the lower image in panel B, but acquired during a different treatment fraction.

A 2D Turbo Spin Echo (TSE) T2-weighted image sequence, acquired interleaved in coronal and sagittal planes, was optimized. A temporal resolution of 0.5 Hz was opted for, which means a sagittal and coronal image within 4 s, since movements of pelvic targets take place over large timescales [11,19]. Different optimization steps have been made and tested on 10 patients with prostate cancer during their MR-Linac treatment. To reduce the saturation band artifacts at the intersection of the imaging stack, a long Repetition Time (TR) was used [17,24]. In addition, compared to the bTFE-cine, a smaller acquisition pixel size of 2 mm and reconstruction pixel size of 0.78 mm was used to improve the spatial localization of the target [25]. The final imaging parameters are reported in the supplementary Table S1 and examples of the image quality of the bTFE-cine and T2-TSE-cine are shown in Fig. 1. This protocol development was performed prior to the evaluation of phase 1 and 2.

### 2.3 Patient selection

Patients treated between January 2023 to January 2024 for prostate, rectum and cervical cancers and LNM were selected for this study. CMM was not yet clinically implemented during this study. A standard Adapt-To-Shape (ATS) workflow was used for these patients. Patients signed informed consent for the prospective Multi-OutcoMe EvaluatioN of radiation Therapy Using the MR-Linac (MOMENTUM) study (NCT04075305) to receive scans for study purposes during treatment, without any treatment time extensions [26]. All scans were acquired during delineation and treatment planning time [26]. In total 52 patients were included in this part of the study.

### 2.4 Phase 1: validation of T2-TSE-cine imaging

**Fig. 2:**
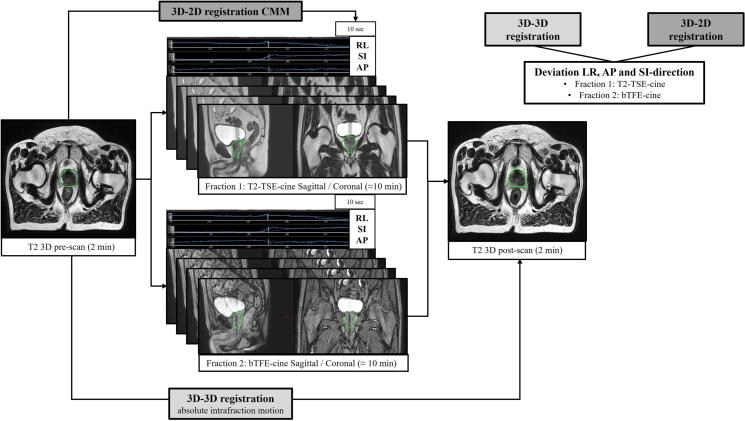
The set-up of the acquisition of 3D and 2D MRI data in phase 1 (only T2-TSE-cine acquisition) and phase 2 (T2-TSE-cine and bTFE-cine). This example is a patient treated for prostate cancer. First, a T2-weighted 3D pre-scan is acquired, followed by 2D interleaved coronal and sagittal cine acquisition and directly afterwards another T2-weighted 3D post-scan is acquired. In phase 2 in fraction 1 the T2-TSE-cine is acquired and in fraction 2 the bTFE-cine is acquired. The 3D-3D registration is performed rigidly and manually using in-house developed software. The 3D-2D registration is calculated automatically in CMM. The average motion of the last 10 s of the 2D images is used to register to the 3D pre-scan. In phase 2 the results of the bTFE-cine are compared to the T2-TSE-cine acquisition, meaning the deviation in LR, AP and SI between the 3D-3D and 3D-2D registration. The 3D-3D registration is used as the absolute intrafraction motion.

First, an evaluation of the T2-TSE-cine sequence was performed. This sequence was acquired in 15 patients treated for prostate cancer and 3 patients treated for cervical cancer during an MR-Linac treatment. First, a T2 3D pre-scan (scan time: 2 min) was acquired, followed by the 2D T2-TSE-cine acquisition (scan time: on average 12.2 min) and directly afterwards another T2 3D post scan (scan time: 2 min) was acquired. The order of acquisitions is depicted in Fig. 2. The time between the pre- and post-scan almost mimics the general beam-on time and is shown in Table 1.

**Table 1:** Overview of the patients, fractions, corresponding dose and acquisition times of all cine images acquired in this work. The average median and range [min–max] of intrafraction motion (IFM) (3D-3D registration) is shown per image acquisition in mm. The average absolute median and maximum deviation per direction (3D-3D vs. 3D-2D registration) is described per image acquisition in mm. T2-cine refers to the T2-TSE-cine image sequence.

	**Phase 1**		**Phase 2**					
**Target**	Prostate CTV	Cervix CTV	Prostate CTV		Rectum CTV		LNM GTV	
Patients (N)	15	3	19		8		6	
Fractions (N)	15	3	38		16		12	
Dose (Gy)	5x7.25	6x6	5x7.25		5x5		5x8 / 3x10	
2D Acquisition mean time and SD (min)	12.4 [±3.2]	12.2 [±3.9]	10.3 [±2.9]		9.6 [±2.7]		7.8 [±2.7]	

	**T2-cine**	**T2-cine**	**bTFE-cine**	**T2-cine**	**bTFE-cine**	**T2-cine**	**bTFE-cine**	**T2-cine**

RL IFM median [min–max] (mm)	0.0 [-2.1–1.6]	0.4 [0–0.7]	0.0 [-1.6–1.5]	0.6 [-1.7–2.4]	−0.3 [-1.7–0.9]	0.2 [-1.1–0.8]	0.5 [-0.6–0.7]	0.1 [-1.8 – 0.9]
AP median IFM [min–max] (mm)	0.6 [-4.9–2.7]	−2.4 [-10.6–0.9]	0.6 [-3.5–6.2]	0.9 [-6.7–6.9]	0.7 [-3.7–2.6]	0.8 [-0.4–3.3]	0.4 [-1.4–1.2]	0.4 [-0.5 – 1.9]
SI median IFM [min–max] (mm)	−0.9 [-3.5–5.8]	0.6 [-1.2–7.4]	0.0 [-3.8–1.3]	−1.0 [-5.2–5.3]	0.0 [-2.3–0.9]	−0.2 [-2.3–0.3]	−0.2 [-0.9–0.9]	−0.4 [-1.3–0]
RL absolute deviation median [max] (mm)	0.3 [0.8]	0.6 [1.9]	0.4 [1.7]	0.5 [1.0]	0.8 [2.0]	0.6 [0.8]	0.4 [0.7]	0.4 [0.7]
AP absolute deviation median [max] (mm)	0.6 [2.5]	0.6 [0.8]	0.6 [4.0]	0.6 [2.7]	0.5 [2.4]	0.4 [0.9]	0.5 [1.4]	0.7 [0.9]
SI absolute deviation median [max] (mm)	0.3 [1.1]	0.6 [0.9]	0.8 [1.9]	0.5 [1.0]	0.9 [1.6]	0.7 [0.9]	0.6 [2.3]	0.6 [1.1]

### 2.5 Phase 2: comparison of T2-TSE-cine and bTFE-cine imaging

Secondly, a comparison of the T2-TSE-cine against the bTFE-cine scan was performed. Twenty patients with prostate cancer containing large intrafraction motion (vector > 3 mm) in the first 3 MR-Linac radiotherapy fractions, were selected for this part of the study. This selection criterion was used to capture patients with likely actionable intrafraction motion amplitude, see for example [27] where a BLS is indicated for motion > 2,5 mm. The same acquisition order was performed as in phase 1 shown in Fig. 2. In one of the five treatment fractions, bTFE-cine images were acquired between the pre- and post-scan and in that same patient in another fraction T2-TSE-cine images were acquired. The same acquisition was performed in six patients having rectum cancer and eight patients with LNM, independent of the amount of intrafraction motion.

### 2.6 Quantitative comparison

The order of acquisitions and registrations is depicted in Fig. 2. The following quantitative comparison was performed for phase 1 (T2-TSE-cine) and phase 2 (bTFE-cine vs. T2-TSE-cine).

#### 2.6.1 3D-3D registration

Rigid registration of the 3D T2-weighted pre- and post-scans was performed around the clinically delineated target volume using in-house developed software, Volumetool [28]. Two experienced Radiation Therapists (RTs), each with over eight years of image registration experience, manually registered the target volumes and visually verified the registrations. This registration, defining the ground truth of the total intrafraction motion, is hereafter referred to as the 3D-3D registration.

#### 2.6.2 3D-2D registration

Intrafraction motion tracking of the clinical target volume (CTV; prostate, rectum, cervix) and the gross tumor volume (GTV; LNM) was evaluated in the 2D-cine images using a research version of the CMM software [7,29,30]. First, CMM uses a template matching algorithm to rigidly register the 2D cine images to the 3D pre-scan within the volume of interest of the defined registration structure (target). This registration can be manually edited by the user. Subsequent cine frames are automatically registered to the multi-2D template using a cross-correlation algorithm [29]. To measure drift motion in relation to the 3D pre scan, the center-of-mass displacement of the registration structure in the LR, AP, and SI directions was averaged over the final 10 s of the 2D cine sequence just prior to the post 3D scan. This time window of 10 s was used to reduce the impact of random fluctuations (i.e. caused by peristalsis). This registration-step is referred to as the 3D-2D registration. These registrations were reviewed and approved by the same two RTs. For small LNMs (GTV < 2 cm^3^), the registration structure was expanded by 1.0 cm around the target to improve registration accuracy.

#### 2.6.3 Deviation analysis: 3D-3D vs 3D-2D

To assess tracking performance in phase 1, the absolute deviation in mm between the 3D-3D and 3D-2D registration was calculated in the LR, AP and SI directions. In phase 2, this analysis was repeated for both the bTFE-cine and T2-TSE-cine acquisitions. For each patient, the median and maximum absolute deviations were calculated per direction. The agreement between both cine sequences measuring the intrafraction motion was visually assessed by a Bland-Altman plot and statistical analysis was performed by a Mann-Whitney *U* test.

#### 2.6.4 Jitter

Additionally in phase 2, the jitter, meaning the positional difference in mm of the registration volume in the SI direction between the sagittal and coronal frames, was measured in the bTFE-cine and T2-TSE-cine per patient. Jitter is an indication of registration uncertainty and in clinical use, CMM will turn off the beam when jitter of > 4 mm is observed. The average jitter and percentages of frames showing jitter larger than 1, 2, 3 and 4 mm respectively are reported.

### 2.7 Visual grading analysis

In phase 2, image quality was assessed using a Visual Grading Analysis (VGA) approach [32]. Five expert observers: 2 Radiation Oncologists, 1 trainee Radiation Oncologist, and 2 RTs with > 5 years of MRI delineation experience, independently evaluated the cine images. All RTs were certified to delineate targets on the MR-Linac [[33], [34]]. Sixteen patients treated for prostate cancer (n = 8), rectum cancer (n = 4) and LNM cancer (n = 4) patients) were randomly selected from phase 2 for VGA. Only sixteen patients were selected because reviewing all patients would be too time consuming for 5 experts. For each patient, two 2-minute cine videos (one bTFE-cine, one T2-TSE-cine) were presented in random order and reviewers were blinded to the sequence type. Image quality was scored on 11 predefined criteria according to a VGA scoring system defined as: 1) not visible, 2) unclear, 3) moderately clear, 4) clear, and 5) very clear. If the structure of interest (e.g. OAR in LNM cases) was not present in the image, the response was omitted. Full scoring criteria are detailed in Supplementary Table S2. In total 32 fractions from 16 patients were rated, which resulted in 160 scores per question. Statistical differences between sequences were analyzed per question using the paired Wilcoxon signed-rank test based on a significance level of p < 0.001.

### 2.8 Measurements of acoustic noise

Due to patient reports of discomfort from bTFE-cine acoustic noise during abdominal CMM treatments on the MR-Linac [16] noise levels of both cine sequences were measured. Recordings were performed using a calibrated Behringer ECM8000 microphone placed in the isocenter. Two sound metrics were evaluated: the peak sound pressure level (LCpeak, in dB(C)) and the equivalent continuous sound level (LAeq, in dB(A)), which reflects average noise over time. Measurements followed ISO/IEC time and frequency weighted standards.

## 3 Results

### 3.1 Quantitative comparison

**Fig. 3:**
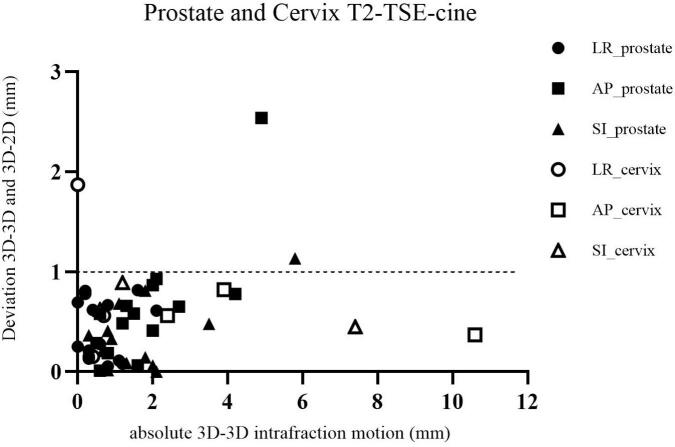
The results of phase 1 of all patients treated for prostate (n = 15) and cervical (n = 3) cancer for the T2-TSE-cine acquisition. The absolute intrafraction motion (3D-3D registration) is presented on the X-axis and the absolute deviation between the 3D-3D and 3D-2D registration is shown on the Y-axis. The black symbols depict the results of the prostate cases and the open symbols the cervix cases. The dashed horizontal line represents a deviation of 1 mm.

The results of the intrafraction motion (3D-3D registration) and deviations between the 3D-3D and 3D-2D registrations of 51 patients and 84 cine acquisitions in phase 1 and 2 are presented in Table 1. One patient (prostate cancer) in phase 2 was excluded due to an incorrectly placed coronal stack in the bTFE-cine acquisition. The absolute intrafraction motion and absolute deviation in LR, AP and SI between the 3D-3D and 3D-2D registration of cervical and prostate cases in phase 1 are presented in Fig. 3.

Fig. 3 represents the comparison in phase 2 between the bTFE-cine and the developed T2-TSE-cine images per treatment site. On average higher deviations between 3D-3D and 3D-2D registrations are shown for the bTFE-cine acquisitions in phase 2, where deviations were calculated in both cine sequences in 33 patients in 3 different directions. In total the bTFE-cine shows 26 out of 99 deviations > 1 mm compared to 6 out of 99 deviations > 1 mm for the T2-TSE-cine. Smaller deviations lead to a better overall agreement in the T2-TSE-cine, however, no statistical significance was observed between the bTFE-cine and T2-TSE-cine deviations and corresponding results can be found in supplementary Table S3 and Fig. S3.

**Fig. 4:**
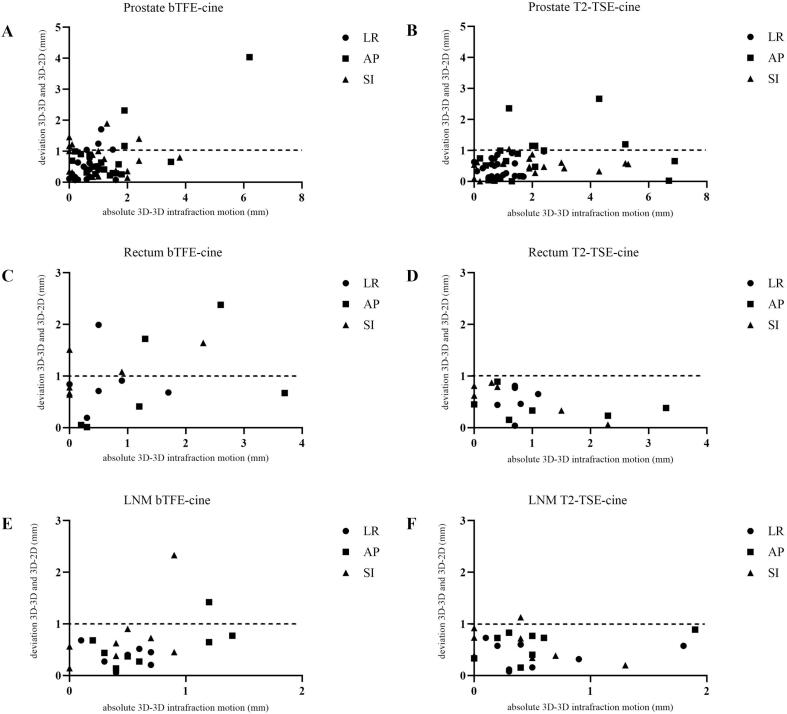
Overview of the absolute intrafraction motion (3D-3D registration) and absolute deviations between the 3D-3D and 3D-2D registration of phase 2 of this study of patients treated for prostate cancer (A-B), Rectum cancer (C-D) and LNM (E-F). The results are shown for the bTFE- and T2-TSE-cine sequence per treatment site and direction. The dashed line represents a deviation of 1 mm. The X- and Y-axes are shown on a different scale per treatment site.

Differences in jitter during phase 2 are presented in Supplementary Fig. S1. In the prostate cases, 23.4 % of frames acquired with the bTFE-cine and 6.1 % of frames acquired with the T2-TSE-cine exhibited > 1 mm jitter. The proportion of frames with > 4 mm jitter was 0.8 % for the bTFE-cine and 0.6 % for the T2-TSE-cine. For patients treated for rectal cancer and LNM cases, the percentage of frames with > 1 mm jitter was 4.9 % and 5.5 % for the bTFE-cine and 12.5 % and 9.7 % for the T2-TSE-cine, respectively. The proportion of frames with > 4 mm of jitter was 0.1 % and 0 % for the bTFE-cine and 1.6 % and 4.5 % for the T2-TSE-cine, respectively (Fig. 4).

### 3.2 Visual grading analysis

**Fig. 5:**
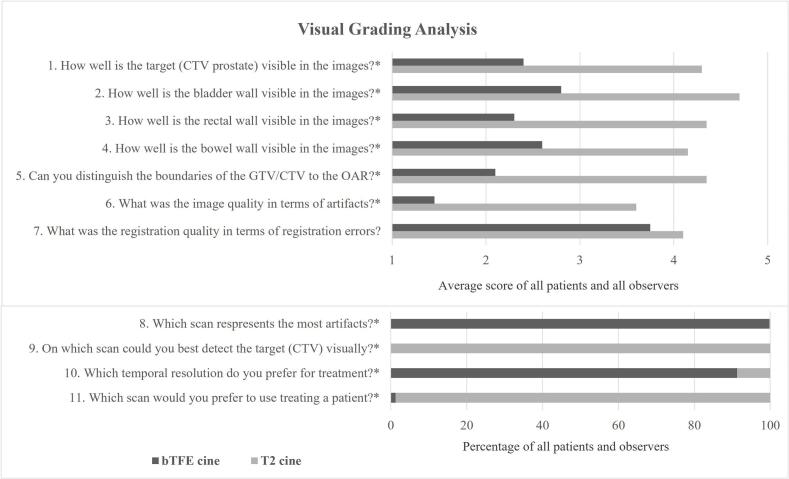
The average results of the Visual Grading Analysis performed within patients treated for prostate (n = 8), rectum (n = 4) and LNM (n = 4) cancer scored by 5 different observers, with in total 160 scores for each question. Questions 1 to 7 are scored from 1) not visible, 2) Unclear, 3) Moderately clear, 4) Clear, and 5) Very clear. Questions 8 to 11 compare both imaging techniques and percentages per technique are presented.* = Statistical significance p < 0.001.

Examples of the VGA can be found in Fig. S2 of the supplementary material. The visual detection for both GTV and CTV was better for the T2-TSE-cine images as they presented superior image contrast and fewer artifacts as presented in Fig. 5. All observers reported higher confidence using T2-TSE-cine. This sequence received significantly higher VGA scores across all questions. Observers found registration errors difficult to detect due to focusing on other evaluation aspects during the two-minute assessment. The temporal resolution of the bTFE-cine was preferred in 91 % of the cases, however, this was not considered to be clinically relevant and observers stated that this was caused by their prior experience in cine images with a high temporal resolution in abdominal cases.

### 3.3 Acoustic noise measurements

A graphical representation of the fast-weighted A-weighted sound levels for both sequences can be found in Fig. S4 of the supplementary material. The LCpeak for the bTFE-cine was 120.0 dB(C) and for the T2-TSE-cine 119.2 dB(C). The equivalent continuous sound level, LAeq, for the bTFE-cine and T2-TSE-cine were 110.7 dB(A) and 100.1 dB(A), respectively.

## 4 Discussion

This study reports the clinical validation of a T2-TSE-cine sequence used for drift compensation in pelvic targets and its comparison to the standard bTFE-cine at the 1.5T Unity MR-Linac using CMM. While the bTFE-cine showed a broader range of deviations between 3D-3D and 3D-2D registrations, the differences were not statistically significant.

[15]. This study provides a quantitative approach as well as a visual analysis by experienced MR-Linac users and in terms of visual assessment a statistically significant preference for the T2-TSE-cine sequence is presented.

A limitation of our study is the fixed 3D-2D-3D acquisition order. In a few patients, movements occurred just before or during the acquisition of the 3D post-scan. Patient motion during 3D post-scans affected registration accuracy in some cases. These deviations were investigated and understood through visual comparison with the last 2D frames.

In phase 1 for the T2-TSE-cine, deviations > 1 mm occurred in 3 of 54 cases, due to intrafraction motion during the 3D post-scans. For example, one prostate case showed blurred post-3D images, leading to > 1 mm deviations in two directions. Another case for cervical cancer missed the target in coronal images due to AP and SI motion, impacting LR registration accuracy as presented in Fig. 3. In phase 2, deviations > 2 mm in both sequences were predominantly associated to rectal movement during 3D scans. However, in one prostate case, bTFE-cine showed a 4.1 mm deviation in AP due to banding artifacts in the prostate causing misinterpretation by the CMM algorithm. Similar issues occurred in an LNM case, having a large deviation of 2,6 mm in SI direction. These artifacts, inherent to bTFE sequences, do not occur in T2-TSE-cine. This highlights that for bTFE-cine scans caution is needed when banding artifacts arise during the scan series as this can lead to incorrect registrations.

Jitter analysis showed higher average jitter in bTFE-cine for prostate cases, including one case > 4 mm jitter due to banding artifacts near a hip implant [34], while in the T2-TSE-cine scan of the same patient this effect was not observed. For rectum and LNM cases, T2-TSE-cine had slightly higher jitter, driven by two outlier cases involving anatomical deformation and out-of-plane motion. Tracking of the GTV in these areas involving a lot of deforming organs is more difficult and more jitter is present.

A limitation of the current CMM workflow is its reliance on 2D-cine images, allowing only translational motion correction. Rotations and deformations, common in pelvic targets due to gas pockets, bowel motion, and bladder filling, remain unaddressed. Further investigation into these scenarios is necessary.

Acoustic noise measurements showed both sequences had comparable peak levels, but T2-TSE-cine showed significantly lower equivalent acoustic noise (9.6 dB less). This aligns with patient reports [16] and suggests T2-TSE-cine improves patient comfort and reduces risk of noise-induced discomfort or hearing damage [31].

Overall, the T2-TSE-cine sequence is a viable alternative to bTFE-cine for MR-guided intrafraction drift correction workflows in pelvic targets. It offers comparable tracking performance, lower acoustic noise levels and improved visual confidence, benefiting both clinicians and patients.

## Funding statement

Funding: None.

## CRediT authorship contribution statement

**Lieke T.C. Meijers:** Writing – original draft, Methodology, Investigation, Project administration, Data curation. **Johannes C.J. de Boer:** Supervision, Conceptualization, Writing – review & editing. **Jochem R.M. van de Voort van Zyp:** Investigation. **Nicole G.P.M. Vissers:** Investigation. **Reijer H.A. Rutgers:** Investigation. **Eveline Alberts:** Conceptualization. **Jasmijn M. Westerhoff:** Formal analysis. **Alice M. Couwenberg:** Investigation. **Marieke I. Snijder-van As:** Resources. **Stefano Mandija:** Resources. **Edwin Versteeg:** Resources. **Martijn P.W. Intven:** Conceptualization. **Bas W. Raaymakers:** Conceptualization. **Astrid L.H.M.W. van Lier:** Conceptualization, Methodology, Visualization, Supervision, Writing – review & editing.

## Declaration of competing interest

The authors declare that they have no known competing financial interests or personal relationships that could have appeared to influence the work reported in this paper.
