# Supplementary material for: Towards clinical implementation of T2-weighted cine imaging for intrafraction drift correction workflows on the 1.5 Tesla magnetic resonance-linear accelerator
Source: Phys Imaging Radiat Oncol. 2025 Nov 1;36:100859. doi: 10.1016/j.phro.2025.100859 (PMC12639456; doi:10.1016/j.phro.2025.100859)
Supplement: Supplementary Data 1 [file mmc1.pdf]

Supplementary material

Sequence	2D bTFE-cine	2D T2-TSE-cine	3D T2-TSE prostate/cervix/rectum/LNM
TR/TE (ms)	3.4/1.71	2000/153	1450/120
Voxel size (mm)	3x3x5	2x2x5	1.5x1.52x.2
Recon Voxel size (mm)	1.3x1.3	0.78x0.78	0.67x0.67x2.0
Flig Angle (°)	40	90	90
NSA factor	1	1*	1
Sense factor	3	2.9	4.5 (CS)
WFS per pix (mm)	0.2	0.197	0.4
Temporal resolution (time per 2 images) (s)	0.424	4.0	-
Actual acquisition time per image (s)	0.212	2.0	-
Shotlength (ms)	-	301	514
K-space dimensions	144x144	180x223	300x300
TFE factor	48	71	91

Table S1*. Imaging parameters of all 2D and 3D imaging sequences used in this study.*
*Another optimization step was performed in this study before Phase 2 since there was no possibility of acquiring cine scans in the Comprehensive Motion Management workflow containing an NSA >1. Instead of using an NSA of 2, a TR of 2000 was implemented in the sequence to reduce the saturation band artifact at the intersection of the imaging stack.

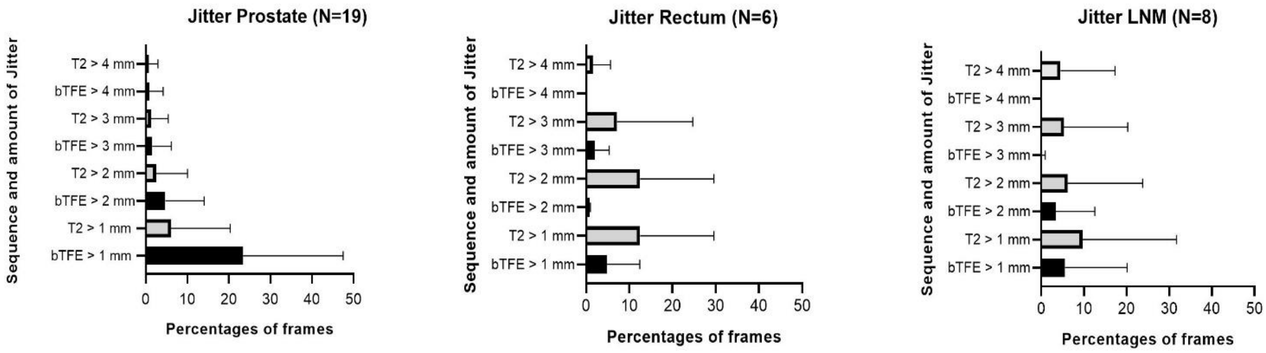

Figure S1. Jitter results are presented as the percentages of frames that show > 1, > 2, > 3, or > 4 mm jitter for patients treated for prostate cancer (A), Rectum cancer (B) and LNM (C). The mean and standard deviation are shown in the graph. T2 refers to the T2-TSE-cine scan.

Score	Definition	Explanation
**1**	Not visible	borders / walls are not at all visible due to bad image contrast / artifacts / resolution
**2**	Unclear	borders / walls are poorly visible due to bad image contrast / artifacts / resolution
**3**	Moderately clear	borders / walls are moderately visible due to average image contrast / artifacts / resolution
**4**	Clear	borders / walls are clearly visible due to good image quality / no artifacts / resolution
**5**	Very clear	borders / walls are extremely well visible due to excellent image quality / no artifacts / resolution

Table S2. *Scoring system for the Visual Grading Analysis. Questions 1 to 7 in the Visual Grading Assessment were scored by 1 – 5 per cine acquisition and patient.*

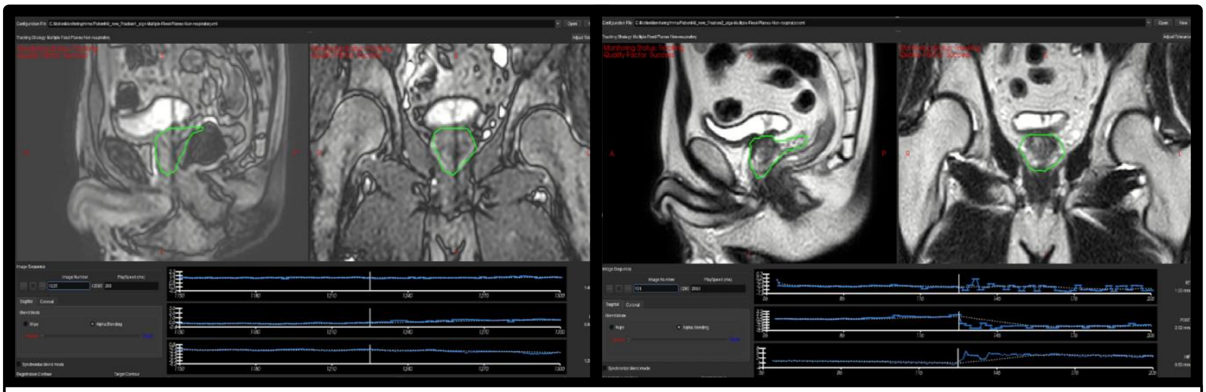

S2a. Prostate CTV. Left image: bTFE-cine. Right image: T2-TSE-cine.

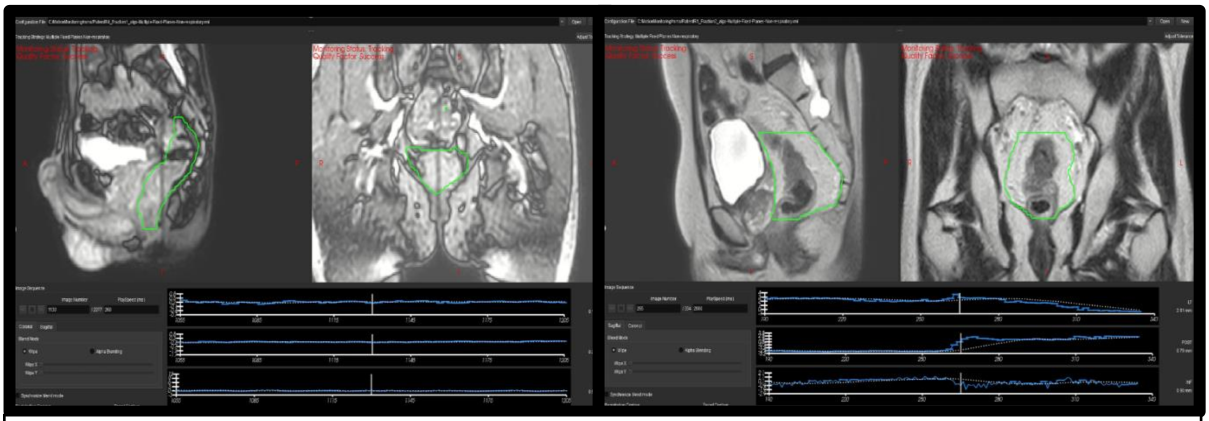

S2b. Rectum CTV. Left image: bTFE-cine. Right image: T2-TSE-cine.

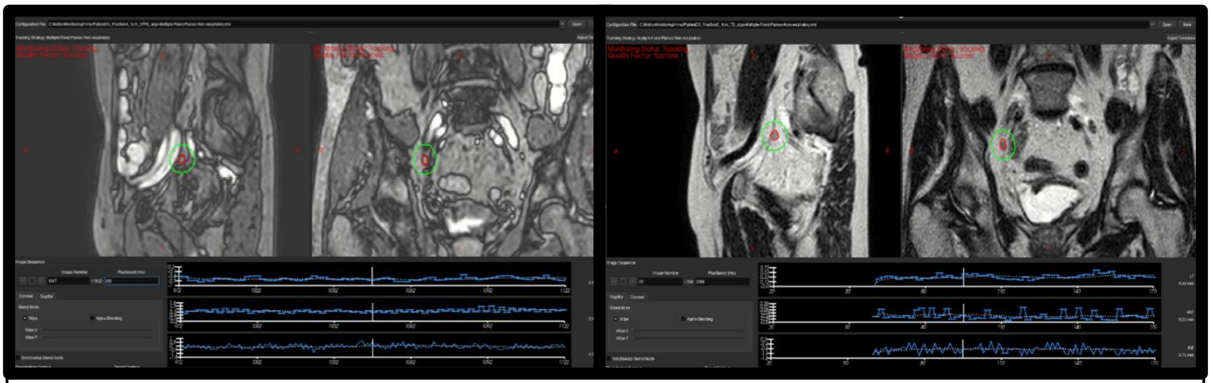

S2c. LNM GTV. Left image: bTFE-cine. Right image: T2-TSE-cine.

Figure S2. *Examples of the Visual Grading Analysis of the different treatment sites: prostate CTV (green), rectum CTV (green) and pelvic lymph node metastasis GTV (red). In the lymph node metastasis patient also the registration structure of a margin of 1.0 cm around the GTV is shown in green. On the left the bTFE-cine images are presented and on the right the T2-TSE-cine images of the same patient. In the bottom the motion trace of the target is shown in LR, AP and SI direction.*

	LR		AP		SI	
**Prostate**	Median (mm)	p-value	Median (mm)	p-value	Median (mm)	p-value
bTFE (N=19)	0.41	0.69	0.58	0.99	0.75	0.25
T2-TSE(N=19)	0.51		0.66		0.56	
**Rectum**						
bTFE (N=6)	0.77	0.15	0.54	0.63	0.93	0.15
T2-TSE (N=6)	0.56		0.35		0.71	
**LNM**						
bTFE (N=8)	0.40	0.75	0.54	0.53	0.92	0.92
T2-TSE (N=8)	0.45		0.73		0.92	

Table S3. *A Mann-Whitney U test was performed to test statistical significance between the median deviation results of the bTFE-cine and T2-TSE-cine. No statistical significance was observed between both cine acquisitions.*

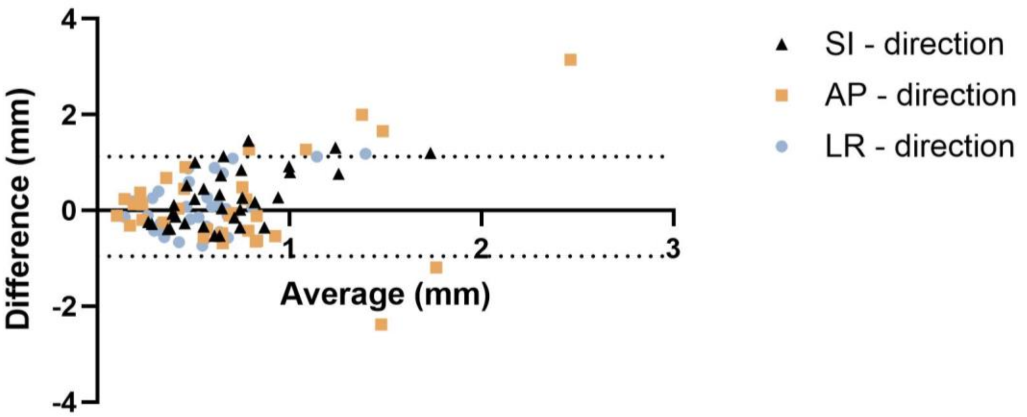

Figure S3. Bland–Altman plot showing the deviations between the bTFE-cine and T2-TSE-cine measurements in the LR, AP and SI directions. Overall, the plot demonstrates a high level of agreement for lower mean values of intrafraction motion. However at higher means, particularly in the AP direction, larger discrepancies are observed. The dashed lines indicate the 95% limits of agreement (LOA).

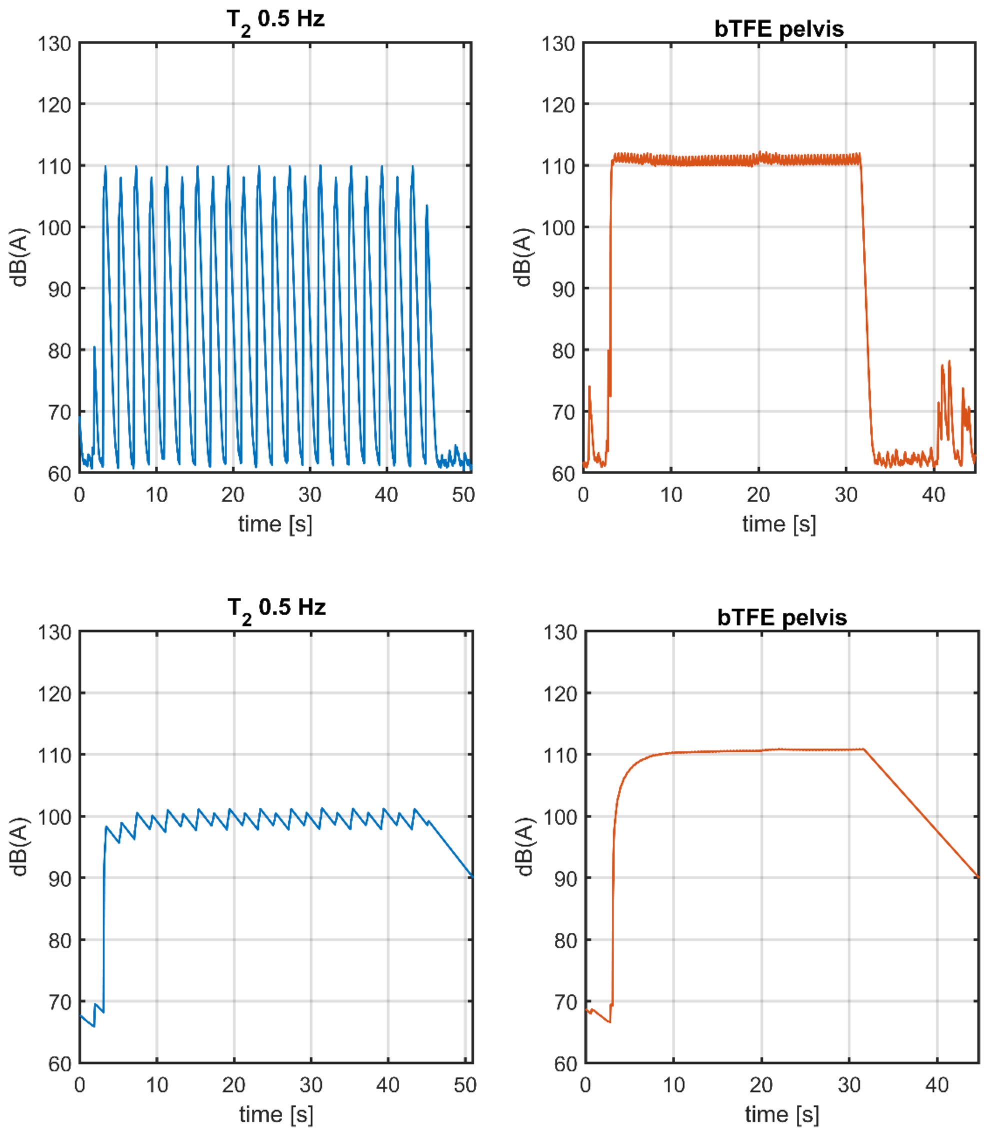

Figure S4. *The results of the acoustic noise experiments measured over time. In blue on the left the measurements of the T2-TSE-cine sequence are presented and on the right in orange the measurements of the bTFE cine. The graphs above depict the acoustic noise measurements with an A-weighted fast time-weighted filter. The different time behavior of both sequences is clearly visible resulting in different calculated LAeq-values. The equivalent continuous sound level, LAeq, for the bTFE-cine and T2-TSE-cine were 110.7 dB(A) and 100.1 dB(A), respectively. The graphs below depict the acoustic noise measurements with an A-weighted slow-time filter.*
